# Genomic characterization of bacteriophage vB_PcaP_PP2 infecting *Pectobacterium carotovorum* subsp. *carotovorum*, a new member of a proposed genus in the subfamily *Autographivirinae*

**DOI:** 10.1007/s00705-017-3349-6

**Published:** 2017-04-13

**Authors:** Jeong-A Lim, Sunggi Heu, Jinwoo Park, Eunjung Roh

**Affiliations:** 1 Microbial Safety Team, National Institute of Agricultural Sciences, Rural Development Administration, Wanju-gun, 55365 Republic of Korea; 2 Research Group of Food Safety, Korea Food Research Institute, Sungnam, 13539 Republic of Korea; 3 Crop Cultivation and Environment Research Division, National Institute of Crop Science, Suwon, 16429 Republic of Korea

## Abstract

Bacteriophage vB_PcaP_PP2 (PP2) is a novel virulent phage that infects the plant-pathogenic bacterium *Pectobacterium carotovorum* subsp. *carotovorum*. PP2 phage has a 41,841-bp double-stranded DNA encoding 47 proteins, and it was identified as a member of the family *Podoviridae* by transmission electron microscopy. Nineteen of its open reading frames (ORFs) show homology to functional proteins, and 28 ORFs have been characterized as hypothetical proteins. PP2 phage is homologous to *Cronobacter* phage vB_CskP_GAP227 and Dev-CD-23823. Based on phylogenetic analysis, PP2 and its homologous bacteriophages form a new group within the subfamily *Autographivirinae* in the family *Podoviridae*, suggesting the need to establish a new genus. No lysogenic-cycle-related genes or bacterial toxins were identified.

**Electronic supplementary material:**

The online version of this article (doi:10.1007/s00705-017-3349-6) contains supplementary material, which is available to authorized users.

*Pectobacterium carotovorum* subsp. *carotovorum* (formerly known as *Erwinia carotovorum* subsp. *carotovora*) is a Gram-negative plant-pathogenic bacterium [12]. It causes soft rot disease in various important crops, such as Chinese cabbage, potato, and lettuce, by producing plant-cell-wall-degrading enzymes. The disease occurs throughout the processing procedure, including production, distribution, and storage, and can cause great economic losses [14].

A bacteriophage is a virus that infects bacteria. Bacteriophages infect only their target bacteria by recognizing a specific receptor on the bacterial surface and destroying them within a short time [7]. Bacteriophages are not harmful to eukaryotes; therefore, they can be applied to reduce complications of disease caused by pathogenic bacteria in humans, animals, and plants. Additionally, food that is contaminated with pathogenic bacteria can be rapidly detected and controlled by bacteriophages [3, 9]. To enlarge the applicability of bacteriophages and overcome their limitations, the genome sequences of various bacteriophages have been analyzed [13]. In cases of bacteriophages infecting *P*. *carotovorum* subsp. *carotovorum*, the genome sequences of only a few bacteriophages have been reported [8, 10]. In this study, the genome sequence of a new bacteriophage, vB_PcaP_PP2 (PP2), which infects *P. carotovorum* subsp. *carotovorum*, was analyzed.

Bacteriophage PP2 was isolated from the soil of a Chinese-cabbage field (Pyeongchang, Korea) where soft-rot disease was occurring. By serial plaque picking, a pure bacteriophage plaque was isolated. After propagation and purification using CsCl gradient ultracentrifugation, bacteriophage PP2 was isolated. *Pectobacterium* isolated from Chinese-cabbage fields where soft-rot diseases had occurred were used for host range determination. The phage DNA was extracted using phenol-chloroform [19] and sequenced on a 454 Genome Sequencer FLX (Roche, Mannheim, Germany). The reads were then assembled into a complete genome sequence using Newbler v2.3 (Macrogen, Seoul, Korea). Open reading frames (ORFs) were predicted using a combination of Glimmer 3[5], GeneMarkS [4], and FgenesB software (Softberry Inc., Mount Kisco, NY). The functions of the ORFs were predicted using BlastP [2] and Pfam domain prediction. Phylogenetic analysis was performed, using MEGA6, by the neighbor-joining and maximum-likelihood methods [16]. The prediction of tRNA coding regions, lifestyle, and the presence of bacterial toxin genes was performed with the tRNAscan-SE program [17], PHACTS program [11], and BTXpred server [15], respectively.

PP2 morphology was observed by transmission electron microscopy (Fig. S1). The phage had the characteristics of a member of the family *Podoviridae*, with a head size of approximately 55 nm and a tail length of approximately 15 nm. It had a 41,841-bp double-stranded DNA with a G+C content of 51.62% and no predicted tRNA. It did not show homology to previously reported *P. carotovorum* subsp. *carotovorum* bacteriophages, although it was more closely related to *Podoviridae* phages PPWS1 and PP2 than the *Siphoviridae* phage My1 and the *Myoviridae* phages PM1 and PM2 (Fig. S2). The PP2 bacteriophage is similar to *Cronobacter* phage vB_CskP_GAP227 and Dev-CD-23823 in terms of its gene composition and amino acid sequence similarity (Fig. 1). Like these two phages, PP2 also contains a single-subunit RNA polymerase gene, which is required for self-replication; thus, it can be classified as a member of the subfamily *Autographivirinae* in the family *Podoviridae* [6]. Thus far, seven genera have been established within the subfamily *Autographivirinae*: *Phikmvvirus*, *Kp32virus*, *Kp34virus*, *Sp6virus*, *Fri1virus*, *Pradovirus*, and *T7virus*. It was proposed previously that vB_CskP_GAP227 and its homologous phages be grouped in a new genus within the subfamily *Autographivirinae* [1]. Phylogenetic analysis of amino acid sequences encoding the RNA polymerase and large terminase (DNA maturase B) could support the proposal of a new genus including PP2, vB_CskP_GAP227, and Dev-CD-23823 (Fig. 2).

**Fig. 1:**
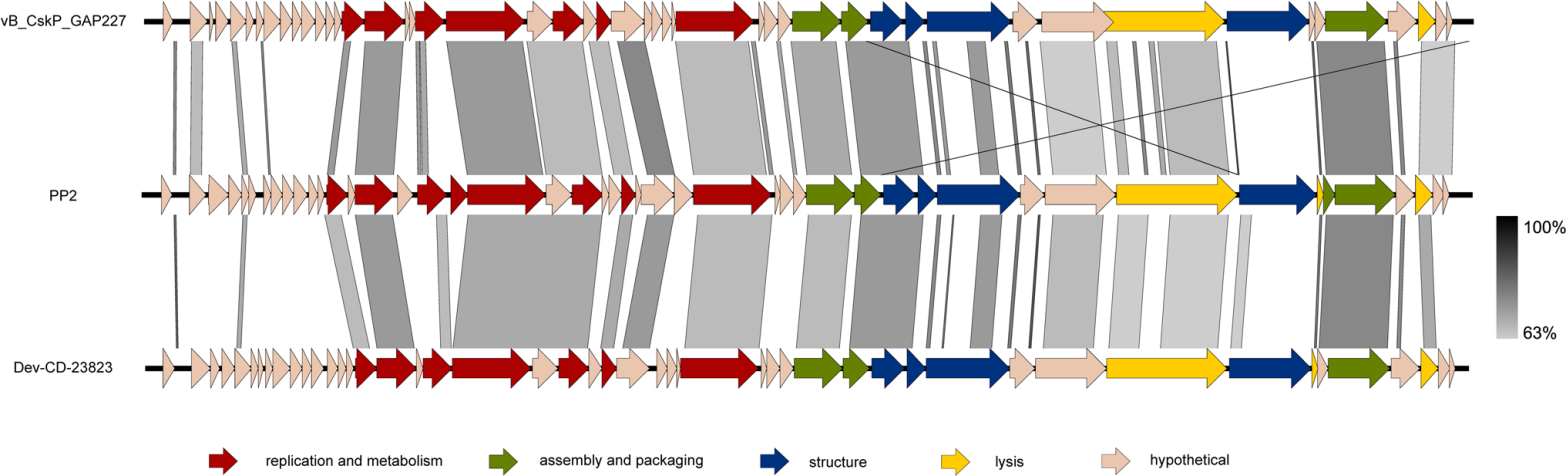
Genome map of bacteriophage PP2 and comparison with homologous phages. Arrows indicate predicted open reading frames responsible for replication and metabolism (red), assembly and packaging (green), structure (blue), and lysis (yellow). Hypothetical proteins are indicated by pink arrows. The amino acid sequence similarities between the phages are indicated by gray shading

**Fig. 2:**
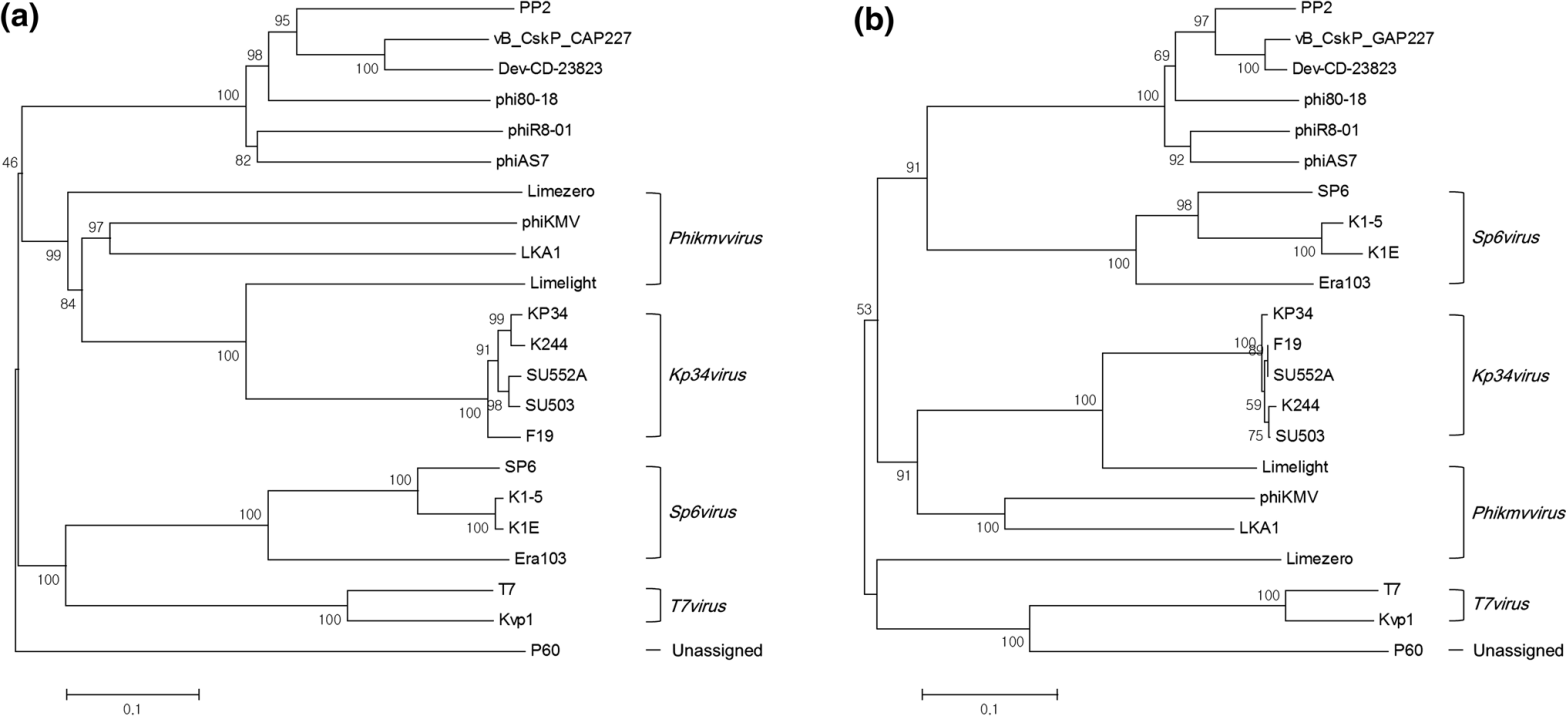
Phylogenetic analyses of the RNA polymerase (A) and terminase large subunit (DNA maturase B) (B) sequences of bacteriophages belonging to the subfamily *Autographivirinae* using the neighbor-joining method. The amino acid sequences were compared using ClustalW. The percentage of replicate trees in which the associated taxa clustered together in the bootstrap test (1000 replicates) is shown next to the branches. The evolutionary distances were computed using the *p*-distance method, and analysis was conducted in MEGA6

Among the 47 predicted ORFs, the functions of 28 were unknown, and 19 could be categorized according to the following functions: replication and metabolism, packaging and assembly, structure, and lysis (Fig. 1 and Table S1). The first group, which includes DNA primase, helicase, ligase, nucleotidyltransferase, RNA polymerase, and exo/endonuclease is needed for replication and metabolism. Scaffolding proteins, DNA maturase, and head-tail connector proteins play a role in virion assembly by forming a procapsid, cutting monomer DNA from concatemers, and joining the head and tail. The predicted structural protein ORFs are major capsid proteins, tail tubular proteins, and tail fiber proteins. Although most genes, except for the hypothetical genes, showed high homology to two bacteriophages (vB_CskP_GAP227 and Dev-CD-23823), there was very low similarity to the tail fiber protein (PP2_040), which is associated with host specificity. PP2 can infect only *P. carotovorum* subsp. *carotovorum* and *P. carotovorum* subsp. *brasiliensis*; *Cronobacter* was resistant to PP2 (Table S2). Related to lysis, lytic transglycosylase, holin and endolysin were predicted. However, there was no protein homologous to spanins, which are accessory lysis proteins of bacteriophages that infect Gram-negative bacteria [18].

The lifestyle (temperate or virulent) of the PP2 bacteriophage could not be predicted using PHACTS software, which may be due to the lack of information on homologous bacteriophages in the database (data not shown). However, there was no lysogenic-cycle-related gene such as a repressor or an integrase. Therefore, we concluded that the PP2 bacteriophage has only a lytic cycle. Lytic bacteriophages have a high chance of bacteria lysis and a low possibility of virulence factor transmission between bacteria. No bacterial toxins were detected in the proteome of the PP2 bacteriophage. Therefore, it has the potential to be used as a safe and useful biocontrol agent.

## Electronic supplementary material

Below is the link to the electronic supplementary material.

Supplementary material 1 (DOC 145 kb)

Supplementary material 2 (DOC 31 kb)

Supplementary material 3 (GBK 85 kb)

Supplementary material 4 (DOC 66 kb)

Supplementary material 5 (DOC 83 kb)
