# Supplementary figures and images for: Genomic characterization of bacteriophage vB_PcaP_PP2 infecting Pectobacterium carotovorum subsp. carotovorum, a new member of a proposed genus in the subfamily Autographivirinae
Source: Arch Virol. 2017 Apr 13;162(8):2441–4. doi: 10.1007/s00705-017-3349-6 (PMC5506502; doi:10.1007/s00705-017-3349-6)

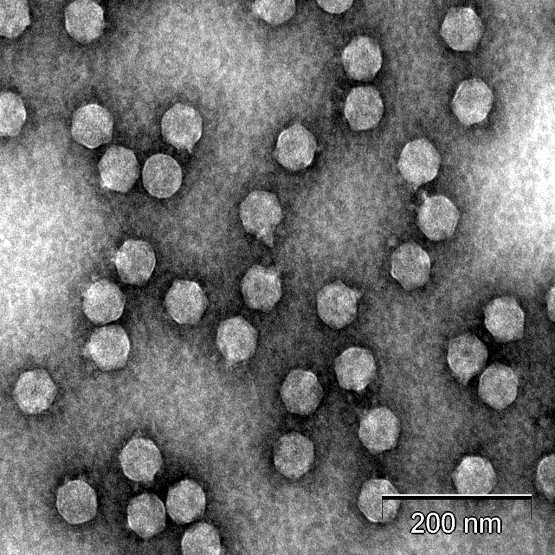


Fig. S1. Electron micrograph of bacteriophage PP2

Supplement: Supplementary file 1 — Supplementary material 1 (DOC 145 kb) [file 705_2017_3349_MOESM1_ESM.doc]
