# Supplementary material for: Genomic characterization of bacteriophage vB_PcaP_PP2 infecting Pectobacterium carotovorum subsp. carotovorum, a new member of a proposed genus in the subfamily Autographivirinae
Source: Arch Virol. 2017 Apr 13;162(8):2441–4. doi: 10.1007/s00705-017-3349-6 (PMC5506502; doi:10.1007/s00705-017-3349-6)
Supplement: Supplementary file 2 — Supplementary material 2 (DOC 31 kb) [file 705_2017_3349_MOESM2_ESM.doc]

Fig. S2. Phylogenetic analysis of the terminase large subunit (DNA maturase B) sequence of *P*. *carotovorum* subsp. *carotovorum* bacteriophages compared to well-known bacteriophages. The amino acid sequences were compared using ClustalW, and the neighbor-joining phylogenetic tree was generated by *P* distance values using MEGA6. The percentage of replicate trees in which the associated taxa clustered together in the bootstrap test (1000 replicates) is shown next to the branches.
