# Supplementary material for: Genomic characterization of bacteriophage vB_PcaP_PP2 infecting Pectobacterium carotovorum subsp. carotovorum, a new member of a proposed genus in the subfamily Autographivirinae
Source: Arch Virol. 2017 Apr 13;162(8):2441–4. doi: 10.1007/s00705-017-3349-6 (PMC5506502; doi:10.1007/s00705-017-3349-6)
Supplement: Supplementary file 4 — Supplementary material 4 (DOC 66 kb) [file 705_2017_3349_MOESM4_ESM.doc]

Table S1. Annotation list of bacteriophage PP2 ORFs

| Locus tag | Start | Stop | Length (aa) | Description |
| --- | --- | --- | --- | --- |
| PP2_001 | 620 | 958 | 112 | hypothetical protein |
| PP2_002 | 1499 | 2032 | 177 | hypothetical protein |
| PP2_003 | 2108 | 2752 | 214 | hypothetical protein |
| PP2_004 | 2740 | 3156 | 138 | hypothetical protein |
| PP2_005 | 3153 | 3374 | 73 | hypothetical protein |
| PP2_006 | 3371 | 3604 | 77 | hypothetical protein |
| PP2_007 | 3840 | 4073 | 77 | hypothetical protein |
| PP2_008 | 4067 | 4435 | 122 | hypothetical protein |
| PP2_009 | 4432 | 4785 | 117 | hypothetical protein |
| PP2_010 | 4782 | 5219 | 145 | hypothetical protein |
| PP2_011 | 5261 | 5542 | 93 | hypothetical protein |
| PP2_012 | 5542 | 5748 | 68 | hypothetical protein |
| PP2_013 | 5819 | 6493 | 224 | DNA primase |
| PP2_014 | 6496 | 6717 | 73 | hypothetical protein |
| PP2_015 | 6710 | 7951 | 413 | DNA helicase |
| PP2_016 | 8043 | 8540 | 165 | hypothetical protein |
| PP2_017 | 8670 | 9599 | 309 | ATP-dependent DNA ligase |
| PP2_018 | 9719 | 10264 | 181 | putative nucleotidyl transferase |
| PP2_019 | 10245 | 12701 | 818 | DNA polymerase |
| PP2_020 | 12714 | 13535 | 273 | hypothetical protein |
| PP2_021 | 13532 | 14518 | 328 | DNA exonuclease |
| PP2_022 | 14476 | 14694 | 72 | hypothetical protein |
| PP2_023 | 14687 | 15097 | 136 | hypothetical protein |
| PP2_024 | 15090 | 15536 | 148 | DNA endonuclease VII |
| PP2_025 | 15533 | 15709 | 58 | hypothetical protein |
| PP2_026 | 15702 | 16751 | 349 | hypothetical protein |
| PP2_027 | 16748 | 17341 | 197 | hypothetical protein |
| PP2_028 | 17351 | 19801 | 816 | DNA-dependent RNA polymerase |
| PP2_029 | 19908 | 20078 | 56 | hypothetical protein |
| PP2_030 | 20075 | 20518 | 147 | hypothetical protein |
| PP2_031 | 20493 | 20894 | 133 | hypothetical protein |
| PP2_032 | 20903 | 22405 | 500 | head-tail connector protein |
| PP2_033 | 22415 | 23242 | 275 | scaffolding protein |
| PP2_034 | 23324 | 24337 | 337 | major capsid protein |
| PP2_035 | 24408 | 25004 | 198 | Tail tubular protein A |
| PP2_036 | 25016 | 27622 | 868 | tail tubular protein B |
| PP2_037 | 27625 | 28392 | 255 | hypothetical protein |
| PP2_038 | 28402 | 30633 | 743 | hypothetical protein |
| PP2_039 | 30645 | 34451 | 1268 | lytic transglycosylase |
| PP2_040 | 34511 | 36943 | 810 | tail fiber protein |
| PP2_041 | 36958 | 37155 | 65 | holin |
| PP2_042 | 37148 | 37522 | 124 | putative DNA maturase A |
| PP2_043 | 37522 | 39417 | 631 | DNA maturase B |
| PP2_044 | 39433 | 40017 | 194 | hypothetical protein |
| PP2_045 | 40050 | 40598 | 182 | endolysin |
| PP2_046 | 40598 | 40957 | 119 | hypothetical protein |
| PP2_047 | 40908 | 41105 | 65 | hypothetical protein |
