# Supplementary material for: Genomic characterization of bacteriophage vB_PcaP_PP2 infecting Pectobacterium carotovorum subsp. carotovorum, a new member of a proposed genus in the subfamily Autographivirinae
Source: Arch Virol. 2017 Apr 13;162(8):2441–4. doi: 10.1007/s00705-017-3349-6 (PMC5506502; doi:10.1007/s00705-017-3349-6)
Supplement: Supplementary file 5 — Supplementary material 5 (DOC 83 kb) [file 705_2017_3349_MOESM5_ESM.doc]

Table S2. Host range of bacteriophage PP2

|  |  |  |  |  |  |  |
| --- | --- | --- | --- | --- | --- | --- |
| **host bacteria** | | **PP2** |  | **host bacteria** | | **PP2** |
| *Pectobacterium carotovorum* subsp. *carotovorum* | isolate 1 | **-** |  | G(-) bacteria /  Enterobacteriaceae | *Cronobacter sakazakii* | **-** |
|  | isolate 2 | **-** |  |  | *Dickeya zeae* | **-** |
|  | isolate 3 | **-** |  |  | *Enterobacter cloacae* | **-** |
|  | isolate 4 | **-** |  |  | *Escherichia coli* O157 | **-** |
|  | isolate 5 | **-** |  |  | *Klebsiella pneumoniae* | **-** |
|  | isolate 6 | **+** |  |  | *Pantoea agglomerans* | **-** |
|  | isolate 7 | **+** |  |  | *Pantoea ananatis* | **-** |
|  | isolate 8 | **+** |  |  | *Rahnella aquatilis* | **-** |
|  | isolate 9 | **-** |  |  | *Salmonella arizonae* | **-** |
|  | isolate 10 | **+** |  |  | *Salnomella typhimurium* | **-** |
|  | isolate 11 | **+** |  |  | *Serratia plymuthica* | **-** |
|  | isolate 12 | **-** |  |  | *Serratia rubidaea* | **-** |
|  | isolate 13 | **-** |  |  | *Shigella boydii* | **-** |
|  | isolate 14 | **+** |  |  | *Shigella flexneri* | **-** |
|  | isolate 15 | **-** |  |  | *Shigella sonnei* | **-** |
|  | isolate 16 | **+** |  |  | *Yersinia enterocolitica* | **-** |
|  | isolate 17 | **-** |  | G(-) bacteria except Enterobacteriaceae | *Acinetobacter calcoaceticus* | **-** |
|  | isolate 18 | **-** |  |  | *Aeromonas hydrophila* | **-** |
|  | isolate 19 | **+** |  |  | *Burkholderia andropogonis* | **-** |
|  | isolate 20 | **+** |  |  | *Burkholderia gladioli* | **-** |
|  | isolate 21 | **-** |  |  | *Chryseobacterium balustinum* | **-** |
| *Pectobacterium carotovorum* subsp. *brasiliensis* | isolate 22 | **+** |  |  | *Neisseria meningitidis* | **-** |
|  | isolate 23 | **+** |  |  | *Pseudomonas chlororaphis* | **-** |
|  | isolate 24 | **-** |  |  | *Pseudomonas corrugata* | **-** |
|  | isolate 25 | **+** |  |  | *Pseudomonas putida* | **-** |
|  | isolate 26 | **+** |  |  | *Pseudomonas syringae* | **-** |
|  | isolate 27 | **+** |  |  | *Stenotrophomonas maltophilia* | **-** |
|  | isolate 28 | **-** |  |  | *Xanthomonas albilineans* | **-** |
| *Pectobacterium carotovorum* subsp. *odoriferum* | isolate 29 | **-** |  |  | *Yersinia enterocolitica* | **-** |
|  | isolate 30 | **-** |  | G(+) bacteria | *Bacillus cereus* | **-** |
|  | isolate 31 | **-** |  |  | *Bacillus megaterium* | **-** |
| *Pectobacterium atrosepticum* | isolate 32 | **-** |  |  | *Bacillus thuringiensis* | **-** |
|  | isolate 33 | **-** |  |  | *Lactobacillus animalis* | **-** |
| *Pectobacterium betavasculorum* | isolate 34 | **-** |  |  | *Listeria innocua* | **-** |
| *Pectobacterium wasabiae* | isolate 35 | **-** |  |  | *Listeria ivanovii* | **-** |
|  | isolate 36 | **-** |  |  | *Listeria monocytogenes* | **-** |
|  | isolate 37 | **-** |  |  | *Staphylococcus xylosus* | **-** |
